# Impact of staying at home measures during COVID-19 pandemic on the lifestyle of Qatar’s population: Perceived changes in diet, physical activity, and body weight

**DOI:** 10.1016/j.pmedr.2021.101545

**Published:** 2021-09-03

**Authors:** Muna Abed Alah, Sami Abdeen, Vahe Kehyayan, Iheb Bougmiza

**Affiliations:** a Community Medicine Department, Hamad Medical Corporation (HMC), Doha, Qatar; b University of Calgary in Qatar, Doha, Qatar; c Community Medicine Department, Primary Health Care Corporation (PHCC), Doha, Qatar; d Community Medicine Department, College of Medicine, Sousse University, Tunisia

**Keywords:** Diet, Physical activity, Weight, Lifestyle, Qatar, COVID-19

## Abstract

Countries worldwide imposed different swift protective measures such as movement restrictions and staying at home measures to mitigate the spread of COVID-19. Despite the benefits gained from such measures in containing the spread of the infection, adverse lifestyle consequences happened. In this study we aimed to assess the impact of staying at home measures imposed during COVID-19 pandemic on dietary behaviors, physical activity, and body weight in Qatar’s population. A population based cross sectional survey was conducted between December 2020 and February 2021 targeting adults ≥18 years. A total of 1408 participants completed the survey in four languages. Concerning the overall diet perception, 27.8%, and 33.2% perceived that their overall diet has become less healthy, or healthier respectively, since the start of staying at home measures. Almost half of all participants reported weight gain. Participants reported increased sitting/reclining time (1.94 h/day mean increase), screen times (2.05 h/day mean increase) with *p* < 0.001, and decreased exercise time (0.11 h/day mean reduction) with *p* < 0.001. The ordinal logistic regression analysis showed that the numbers of unhealthy dietary behaviors adopted by participants, and adverse changes in exercise, setting/reclining and screen times were significantly associated with weight gain. The results of this study indicate that peoples in Qatar have experienced adverse lifestyle changes with regard to their diet, physical activity, and body weight. More emphasis should be put on encouraging people to maintain healthy lifestyle behaviors during home confinement measures that might be imposed during any public health crises or any potential future outbreaks.

## Highlights

• Half of participants perceived some weight gain during staying at home measures.
• One third perceived that their overall diet became less healthy with home confinement.
• The reduction in exercise time was significantly associated with weight gain.
• Increase in setting/reclining, and screen times were significantly associated with weight gain.

## 1 Introduction

The COVID-19 pandemic has caused governments in various countries to take swift and protective measures to limit the spread of the infection by closing shopping malls, gyms, and schools, and discouraging social gatherings. Many countries resorted to adopting more strict measures such as imposing total lockdowns or even curfews (Abed Alah et al., 2019). Unfortunately, such mandated restrictions on movements prevented people from going to work, school, or gym, and forced them toward more sedentary lifestyles (Stockwell et al., 2021, Violant-Holz et al., 2020). The evolving literature has shown reductions in physical activity among populations in various countries due to lockdowns (Stockwell et al., 2021, Ammar et al., 2020, Meiring et al., 2021, Robinson et al., 2021). Some studies reported some mode-specific increases in physical activity particularly among subgroups who were less active prior lockdowns. However, the total physical activity levels in these respective populations still decreased (Stockwell et al., 2021, Suka et al., 2021, Maugeri et al., 2020 Jun 1, Schnitzer et al., 2020 Aug). A sizable body of evidence has suggested that sedentary behaviors are independent risk factors for adverse health conditions. Such behaviors are associated with increased cardiovascular and all-cause mortality (Seguin et al., 2014, Dunstan et al., 2010, Kim et al., 2013, George et al., 2013, León-Muñoz et al., 2013), type 2 diabetes mellites (Krishnan et al., 2009, Ford et al., 2010, van der Berg et al., 2016), cancer (Campbell et al., 2013, Patel et al., 2010, Seguin et al., 2014, Dunstan et al., 2010, Kim et al., 2013), adverse metabolic outcomes, and increased incidence of metabolic syndrome (van der Berg et al., 2016, Mabry et al., 2012, Xiao et al., 2016, Healy et al., 2008, Thorp et al., 2010, Ford et al., 2010, Mitchell et al., 2018). Moreover, physical inactivity can alter glucose and insulin metabolism and impair skeletal muscle protein synthesis (Bowden Davies et al., 2019, Oikawa et al., 2019). It can also adversely affect mental health and result in detrimental psychological outcomes (Violant-Holz et al., 2020).

Movement restrictions and the temporary closure of businesses and shifting to working from home during the COVID-19 pandemic may affect normal food-related practices (World Health Organization, 2020). The closure of food suppliers and the limited access to fresh food that resulted from closure of grocery retailers, limited delivery services, and panic buying behaviors exhibited by some people (Leone et al., 2020), may result in an increased consumption of highly processed foods, which tend to be high in fats, sugars and salt (World Health Organization, 2020). Feelings of boredom resulting from staying at home for prolonged periods of time may increase the desire to eat unhealthy snacks as opposed to eating healthy foods (Moynihan et al., 2015). Boredom has been associated with an increased energy intake as well as the consumption of higher quantities of fats, carbohydrates, and proteins (Moynihan et al., 2015). The stress surrounding this pandemic has pushed people toward overeating, mostly looking for sweetened “comfort foods” (Yılmaz and Gökmen, 2020, Razzoli et al., 2017). A growing body of evidence has shown that staying at home measures during the COVID-19 pandemic has had a negative impact on several aspects of lifestyles such as levels of physical activity and dietary behaviors in health-compromising directions. Studies have shown that during the lockdown associated with the pandemic people reported less physical activity (Schnitzer et al., 2020 Aug, Ammar et al., 2020, Meiring et al., 2021, Robinson et al., 2021, Ismail et al., 2020, Husain and Ashkanani, 2020, Zachary et al., 2020, Górnicka et al., 2020), weight gain (Zachary et al., 2020), and more unhealthy dietary patterns such as snacking more frequently, eating out of control, and eating more (Ammar et al., 2020, Robinson et al., 2021, Ismail et al., 2020, Husain and Ashkanani, 2020, Zachary et al., 2020), and an increase in daily sitting and screen times (time spent in front of TV, smart devices, laptops) (Ammar et al., 2020, Ismail et al., 2020, Husain and Ashkanani, 2020, Górnicka et al., 2020).

In Qatar, maintaining healthy lifestyles is a priority as reflected in the government’s public health strategy 2017–2022 (Ministry of Public Health Qatar, 2021). Six wellness centers were established in Primary Health Care to offer wellness services such as weight control, exercise regimens, and dietary modifications by healthy lifestyle specialists (Primary Healthcare Corporation, 2021). The ultimate goal of these centers is the prevention and control of any existing lifestyle related diseases (Primary Healthcare Corporation, 2021). Unfortunately, such services were interrupted during the pandemic in an attempt to mitigate the spread of the infection. Qatar implemented several preventive and restrictive measures beginning March 2020 to contain the spread of COVID-19. These measures included limiting the number of people going out to work to 20% of total employees, closure of educational institutions, shopping malls, wedding halls, gyms, parks, beaches, and restaurants, interruption of some public transportation services, and strongly emphasizing the importance of staying at home through various media channels. However, Qatar did not impose a complete lockdown or a curfew; people were still allowed to leave their homes whenever they wanted, and they were able to practice outdoor physical exercises. However, with the other restrictive measures in place and with the hot humid weather in Qatar during that period, people were reluctant to leave their homes unless absolutely necessary. Easing and lifting of restrictions took place over a phased controlled process that involved four phases with first phase beginning on June 15, 2020 and the fourth on September 15, 2020, with two weeks interval between each of the phases (Supreme Committee for Crisis Manegment, 2020). The transition from one phase to the next depended on a stringently defined “monitor-review-adapt” process and close monitoring of a set of restriction-specific key performance indicators such as the number of new COVID-19 cases and the epidemic curve (Supreme Committee for Crisis Manegment, 2020). Based on these factors, Qatar could not reach phase four on time for returning to normal. In fact, at the time of conducting this study between December 2020 and February 2021, while most restrictions were eased, they never reached a back to full normal state with 100% operational services. People still worked from home, restaurants, gyms, shops, and malls opened with limited capacities, and staying at home recommendations continued to be emphasized. We believe that assessing the impact of such restrictive measures on the lifestyle of the multicultural population of Qatar that hosts over 80 different nationalities is indeed a fertile area for research that needs to be explored.

In this population-based study we aimed to assess the impact of staying at home measures imposed during the COVID-19 pandemic on dietary behaviors, physical activity, and body weight. To the best of our knowledge this is one of the few studies to address this area in the Middle East, which will add value to the preexisting and evolving literature. Our objectives were to explore the changes reported by Qatar’s population in their dietary behaviors, physical activity, and body weight and their associated factors. We believe that the results of this study will promote the availability of evidence for policy makers and will guide the implementation of effective lifestyle related interventions.

## 2 Methods

### 2.1 Study design and the target population

A population based cross-sectional survey was conducted between December 2020 and February 2021. The target population included adults ≥18 years, who stayed in Qatar for at least two months between March and August 2020, which was the time when Qatar had strict public health measures such as closure of shopping malls, schools, gyms, and working from home, and emphasized the importance of staying at home measures.

### 2.2 Study procedure

A web-based self-administered survey was developed using the SurveyMonkey software. The link to the survey was posted on the social media platforms of Hamad Medical Corporation (HMC) such as Instagram, Facebook, and Twitter, which are generally accessible by the public. We also utilized snowballing sampling method by circulating the link through emails and WhatsApp groups. The survey started with an introductory letter that stated the purpose of the study, that participation was voluntary, and assured the anonymity and confidentiality of collected information. Taking the survey implied informed consent. Participants were free to terminate the survey at any time. Reminders with reposting of the links on social media were done on a regular basis. Ethical approval was obtained from the Institutional Review Board (IRB) of HMC (MRC 05-195).

### 2.3 Study questionnaire

The questionnaire was developed in English after a thorough literature review and after consulting with experts in the field. It was then translated into three other languages (Arabic, Malayalam, and Urdu) by an accredited translation body. We conducted a comprehensive literature review using databases like PubMed, and Google Scholar utilizing different sets of keywords and Medical Subject Headings (MeSH) terms. We consulted a nutritionist and a lifestyle specialist upon selecting the final items to be included in the questionnaire who also assured the face and content validities of the questionnaire and modified the items to avoid ambiguity. The questionnaire consisted of three sections. The first section explored participants’ sociodemographic characteristics (age, gender, nationality, marital status, highest degree of education, employment status and whether they worked from home or not during the staying at home measures, and the presence of any chronic diseases). The second and the third sections explored the changes in dietary behaviors and their underlying factors in addition to changes in weight, and changes in physical activity and sedentary behaviors (including changes in time spent in exercise, sitting/reclining, and screen time) respectively.

### 2.4 Outcome measures

We measured participants’ self-reported perceived changes in their dietary behaviors and physical activities. To assess changes in diet, participants were asked to select the type of dietary changes they adopted since the start of COVID-19-related staying at home measures for the period between March and August 2020 using seven statements (five statements indicated unhealthy changes and two indicated healthier ones). The statements were: “I tend to eat more fatty food”, “I tend to eat more sugar/chocolate/sweets”, “I tend to eat more fast/junk food”, “I tend to eat more processed/canned food than fresh food”, “I tend to eat larger quantities of food”, “I tend to eat more vegetables/fruit and fresh food”, and “I tend to depend more on home cooking”. The total number of unhealthy dietary behaviors was calculated for each participant. Reasons for the underlying dietary changes were assessed for each direction of change by allowing participants to select one or more reasons from a list that was developed after thorough literature review, and they were allowed to specify other reasons not present in the list. Examples for reasons behind unhealthy changes included: “Increase stress and worry during COVID-19 pandemic makes me eat more”, “Increased boredom while staying at home makes me eats more., “Financial problems since the start of COVID-19 related measures limited my ability to buy healthier alternatives”, “Processed/canned food is easier to store and use than fresh food during “staying at home” measures”. Examples of reasons behind healthier changes included: “Healthy eating habits strengthen my immunity against contracting COVID-19”, “Less accessibility to fast food restaurants”, “Closing of gyms and movement restriction make me more worried about my fitness and or weight and so I tend to eat healthier alternatives”. Participants also reported their perceived weight change on an ordinal scale indicating their average weight gain since the start of staying at home measures (no change, <3 kg, 3–6 kg, 7–10 kg, >10 kg). To assess the changes in physical activity and sedentary behaviors, participants were asked to report separately the average time spent in exercise (regardless of the type or intensity of exercise), sitting/reclining, and screen time expressed as hours/day both before and during the staying at home measures. They were also asked whether they used to go to the gym regularly before the gym closures.

### 2.5 Statistical analysis

Data analysis was performed using the IBM SPSS Statistics for Windows, Version 26.0. Armonk, NY: IBM Corp. Descriptive statistics were presented as frequencies and percentages for categorical variables. After testing for normality and taking into account the number of unhealthy dietary behavioral changes as an ordinal dependent variable, the nonparametric Mann-Whitney *U* test and Kruskal-Wallis test were applied to compare the number of unhealthy changes between two or more groups. The Wilcoxon Signed Rank test was used to test the differences in the times spent in exercise and in sitting/reclining, and screen time before and during the staying at home measures. Rank biserial correlation was calculated to measure the effect size for these comparisons (small 0.10−<0.30, medium 0.30−<0.50, large ≥ 0.50). Chi-square test was used to determine the differences between categorical variables. To determine potential predictors for decreased exercise time and increased sitting/reclining, and screen times, a multivariable logistic regression analysis was executed. To determine potential predictors for weight changes, an ordinal logistic regression analysis was performed. The associations between risk factors and outcomes were presented as adjusted odds ratios (ORs) and 95% confidence intervals (95%CIs). Goodness of Fit was assessed using Hosmer Lemeshow test for logistic regression, and Pearson and Deviance tests for ordinal regression. *P*-values less than 0.05 were considered significant.

## 3 Results

### 3.1 Sociodemographic characteristics

As shown in Table 1, the survey was completed by 1408 participants. Of these, 825 (58.6%) completed the English version, followed by 23.2%, 17.3%, and 0.9% of the participants completed the Malayalam, Arabic, and Urdu versions, respectively. Male to female ratio was 1.4:1 and 58.8% were males. The majority of participants 1084 (77%) were 25–44 years old, 1132 (80.4%) were married, and 1107 (78.6%) had completed a college degree or higher. The top three nationalities were Indians (53.4%), Filipino (5.2%), and Qatari (4.3%). Almost three quarters (1070; 76%) were employed and 53.1% of them were working from home during staying at home measures. Of the total participants, 306 (21.7%) suffered from chronic diseases.

**Table 1:** Sociodemographic profiles and background information of the participants.

Variable		No (%)
Age	18–24	57 (4.0)
	25–34	540 (38.4)
	35–44	544 (38.6)
	45–54	203 (14.4)
	55–64	59 (4.2)
	65+	5 (0.4)
Gender	Male	828 (58.8)
	Female	580 (41.2)
Nationality (classification by regions)*	Americas	45 (3.2)
	Sub-Saharan Africa	54 (3.8)
	Europe	117 (8.3)
	Middle East – North Africa	273 (19.4)
	Asia – Pacific	919 (65.3)
Highest degree of education	No formal education	18 (1.3)
	High school diploma	228 (16.2)
	College or Higher	1107 (78.6)
	Vocational training	55 (3.9)
Marital status	Married	1132 (80.4)
	Not married	276 (19.6)
Presence of chronic disease/s^†^	Yes	306 (21.7)
	No	1102 (78.3)
Employment related information		
Employment status	Employed	1070 (76.0)
	Not employed	338 (24.0)
Nature of work^‡^	Mostly office work	746 (69.7)
	Mostly field work	324 (30.3)
Working from home as part of “staying at home” measures^‡^	Yes	568 (53.1)
	No	502 (46.9)
Collective duration (either consecutively or intermittently) of working from home as part of “staying at home” measures^§^	Less than one month	84 (14.8)
	1 – 2 months	109 (19.2)
	3–4 months	168 (29.6)
	5 months or more	207 (36.4)

* >50 different nationalities were reported.

† Most commonly reported chronic diseases were: Diabetes, Hypertension, Asthma and Cardiovascular diseases.

‡ Denominator is the number of employed participants (n = 1070).

§ Denominator is the number of those working from home during “staying at home measures (n = 568).

### 3.2 Changes in dietary behaviors

**Fig. 1:**
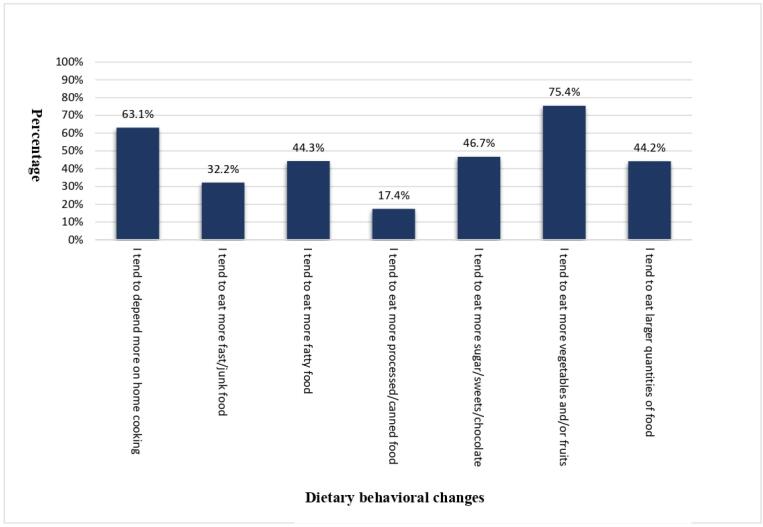
Common dietary behavioral changes reported by participants during COVID 19 related staying at home measures.

Concerning the overall perception of diet, about one third (392, 27.8%) of participants perceived that their overall diet had become less healthy since the start of staying at home measures; 468 (33.2%) perceived a healthier change, and the remaining perceived no changes in their overall diet. As shown in Fig. 1, participants reported several unhealthy dietary behavioral changes such as consuming more fatty food (44.3%), sugar/sweets (46.7%), junk food (32.2%), processed and/or canned food (17.4%), and larger quantities of food (44.2%). On the other hand, 75.4% and 63.1% of them reported consuming more vegetables, fruits, and fresh food and depending more on home-cooking respectively. However, most participants who reported such healthy dietary behaviors also reported at least one unhealthy one. For example, 65.9% and 68.8% of those who reported consuming more fruits and vegetables, and depended more on home cooking, reported one or more unhealthy dietary changes, respectively. Participants who perceived unhealthy changes in their overall diet attributed these changes mostly to feelings of boredom during staying at home measures (284, 72.4%), longer time spent in front of screens such as TV, mobiles, computers (274, 69.9%), and stress and worry during the COVID-19 pandemic (231, 58.9%). A smaller proportion reported other factors such as limited accessibility and/or availability of healthier food alternatives (95, 24.2%), that processed/canned food was easier to store and use than fresh food during staying at home measures (79, 20.2%), and inability to buy healthier alternatives due to COVID-19 related financial constraints (76, 19.4%). On the other hand, healthier dietary changes among those who perceived a healthier overall diet were attributed to participants’ beliefs that a healthier diet would strengthen their immunity against contracting the COVID-19 infection (373, 79.7%), and fear of catching the infection if ordered food from restaurants (226, 48.3%). Participants in the younger age groups, females, unmarried, and those working from home reported significantly higher numbers of unhealthy dietary changes. Participants with a history of chronic diseases and with nationalities of Asia-Pacific origin had significantly lower number of unhealthy dietary changes Table 2.

**Table 2:** Differences in the number of unhealthy dietary behaviors adopted by participants among different sociodemographic subgroups during “staying at home” measures.

Variable			Median number of the adopted unhealthy dietary behaviors	*p*-value
Age		18–24	3	**<0.001**
		25–34	2	
		35–44	1	
		45–54	1	
		55–64	0	
		65+	0	
Gender		Male	1	**0.03**
		Female	2	
Nationality (classification by regions)		Americas	2	**<0.001**
		Sub-Saharan Africa	2	
		Europe	2	
		Middle East – North Africa	2	
		Asia – Pacific	1	
Highest degree of education		No formal education	2	0.91
		High school diploma	1	
		Vocational training	2	
		Collage or higher	2	
Employment status during “staying at home” measures	Employed	Converted to working from home	2	**0.003**
		Continued working regularly	1	
		Not employed	2	
Marital status		Married	1	**<0.001**
		Unmarried	2	
Presence of chronic disease/s		Yes	1	**0.042**
		No	2	

### 3.3 Changes in physical activity

Participants reported a minor, yet statistically significant reduction in the average time spent in exercise (0.11 h/day). This equals to 6.6 min/day mean reduction, *p* < 0.001 with small effect size (r −0.17). However, group analysis demonstrated in Table 3 showed a statistically significant reduction in exercise time for those who used to go to gyms before their closure (*p* < 0.001) and and minor non significant increase for those who did not (p = 0.527). Participants also reported a significant increase in sitting/reclining time (1.94 h/day mean increase), screen time (2.05 h/day mean increase) with *p* < 0.001 and large effect sizes (r 0.80, and 0.76 respectively).

**Table 3:** Changes in exercise, setting/reclining, and screen times before and during “staying at home” measures.

Variable		Mean difference*	T0 Median (Q1-Q3)	T1 Median (Q1-Q3)	*p-*value^†^	*r*^‡^
Exercise time (Hours/day)	Previously active (attended gym regularly)	−0.57	1 (1–1)	1 (0–1)	**<0.001**	−0.60
	Previously not active	0.02	1 (1–1)	1 (1–1)	0.527	
Setting/reclining time (Hours/day)		1.94	3 (2–6)	6 (3–10)	**<0.001**	0.76
Screen time (Hours/day)		2.05	3 (2–6)	6 (3–9)	**<0.001**	0.80

Abbreviations: T0, time before “staying at home” measures; T1 time during “staying at home” measures; Q1, quartile 1 (25th percentile); Q3, quartile 3 (75th percentile); *r*, Rank-biserial correlation coefficient.

* The mean difference in the exercise time (hours/day) for the whole sample (regardless of their previous gym attendance) was −0.11 (hours/day).

† Using Wilcoxon signed rank-rest.

‡ When applying Wilcoxon signed rank-rest for the difference in the exercise time (hours/day) before and during “staying at home” measures for the whole sample (regardless of their previous gym attendance), there was significant difference with p < 0.001 and effect size *r* - 0.17.

To determine the predictors of changes in exercise, sitting/reclining and screen times, we carried out three models of multivariable logistic regression considering the change (increase or no increase, decrease or no decrease) in times as a binary dependent variable. The three models were statistically significant when compared to the null models (*p* < 0.001) and of good fit. Regression analysis showed that participants with nationalities of Middle Eastern-North African and European origins were about two times more likely to report an increase in sitting/reclining and screen times compared to those of Asia-pacific origin (*p* < 0.001). Those who were previously active and used to go to the gym regularly were more likely to report adverse changes in all of the measured times compared to the less active people. Table 4 shows the results of the logistic regression analysis.

**Table 4:** Determinants of changes of exercise, sitting/reclining and screen times using Chi square test and multivariable logistic regression analysis.

Variable			Exercise Time				Sitting/Reclining Time				Screen Time			
			Decreased No (%)	***χ*^2^ test***p*-value	**Multivariable regression analysis**		Increased No (%)	***χ*^2^ test***p*-value	**Multivariable regression analysis**		**Increased**No (%)	***χ*^2^ test***p*-value	**Multivariable regression analysis**	
					AOR (95%CI)	*p*-value			AOR (95%CI)	*p*-value			AOR (95%CI)	*p*-value
**Age**	18–24		21 (36.8)	0.258	1 [Reference]		43 (75.4)	**0.018**	1 [Reference]		47 (82.5)	**0.031**	1 [Reference]	
	25–34		157 (29.1)		0.82 (0.42–1.62)	0.574	331 (61.3)		0.65 (0.32–1.32)	0.231	357 (66.1)		0.45 (0.23–1.10)	0.082
	35–44		135 (24.8)		0.68 (0.34–1.37)	0.278	308 (56.6)		0.53 (0.26–1.09)	0.083	344 (36.2)		0.43 (0.19–0.96)	**0.038**
	45–54		51 (25.1)		0.58 (0.27–1.27)	0.174	116 (57.1)		0.12 (0.24–1.11)	0.091	120 (59.1)		0.34 (0.15–0.80)	**0.012**
	55–64		13 (22.0)		0.45 (0.17–1.14)	0.092	31 (52.5)		0.42 (0.17–1.01)	0.054	35 (59.3)		0.34 (0.13–0.90)	**0.030**
	65+		1 (20.0)		0.58 (0.56–5.95)	0.643	5 (100.0)		0.00	0.999	3 (60.0)		0.29 (0.04–2.19)	0.229
**Gender**	Male		198 (23.9)	**0.003**	0.86 (0.64–1.16)	0.326	459 (55.4)	**0.001**	0.98 (0.74–1.29)	0.877	493 (59.5)	**<0.001**	0.87 (0.65–1.15)	0.236
	Female		180 (31.0)		1 [Reference]		375 (64.7)		1 [Reference]		413 (71.2)		1 [Reference]	
**Nationality (classification by regions)**	Americas		17 (37.8)	**<0.001**	1.31 (0.66–2.58)	0.442	30 (66.7)	**<0.001**	1.30 (0.67–2.54)	0.440	32 (71.1)	**<0.001**	1.22 (0.61–2.46)	0.571
	Sub-Saharan Africa		18 (39.3)		1.40 (0.75–2.60_	0.291	39 (72.2)		2.11 (1.13–3.97)	**0.020**	40 (74.1)		1.89 (0.99–3.60)	0.054
	Europe		46 (34.8)		1.46 (0.92–2.32)	0.113	91 (77.8)		2.41 (1.47–3.95)	<**0.001**	94 (80.3)		2.12 (1.27–3.54)	**0.004**
	Middle East – North Africa		95 (22.0)		1.34 (0.97–1.85)	0.072	195 (71.4)		1.94 (1.42–2.65)	<**0.001**	214 (78.4)		2.27 (1.63–3.17)	**<0.001**
	Asia – Pacific		202 (27.8)		1 [Reference]		479 (52.1)		1 [Reference]		526 (57.2)		1 [Reference]	
**Highest degree of education**	No formal education		5 (2.78)	**0.004**	2.46 (0.64–9.48)	0.191	9 (50.0)	**0.011**	0.84 (0.27–2.64)	0.766	10 (55.6)	**0.002**	0.70 (0.22–(2.19)	0.535
	High school diploma		44 (19.3)		1.55 (0.66–3.65)	0.319	116 (50.9)		0.93 (0.50–1.74)	0.830	122 (53.5)		0.60 (0.31–1.13)	0.113
	College or Higher		321 (29.0)		2.23 (1.01–4.95)	**0.049**	681 (61.5)		1.19 (0.67–2.11)	0.548	739 (66.8)		0.83 (0.46–1.50)	0.533
	Vocational training		8 (14.5)		1 [Reference]		28 (50.9)		1 [Reference]		35 (63.6)		1 [Reference]	
**Employment status**	Employed	Work from home	160 (28.2)	**0.015**	0.96 (067–1.37)	0.813	379 (66.7)	**<0.001**	1.24 (0.89–1.74)	0.206	406 (71.5)	**<0.001**	1.23 (0.86–1.75)	0.258
		Work regularly	113 (22.5)		0.81 (0.54–1.20)	0.283	236 (47.0)		0.60 (0.42–0.85)	**0.004**	257 (51.2)		0.55 (0.38–0.80)	**0.001**
	Not employed		105 (31.1)		1 [Reference]		219 (64.8)		1 [Reference]		243 (71.9)		1 [Reference]	
**Marital status**	Married		282 (24.9)	**0.001**	0.89 (0.64–1.25)	0.496	645 (57.0)	**<0.001**	0.85 (0.62–1.18)	0.342	708 (62.5)	**0.004**	0.99 (0.71–140)	0.989
	Not married		96 (34.8)		1 [Reference]		189 (68.5)		1 [Reference]		198 (71.7)		1 [Reference]	
**Chronic disease**	Yes		84 (27.5)	0.787	1.23 (0.89–1.69)	0.211	170 (55.6)	0.139	0.92 (0.69–1.23)	0.577	187 (61.1)	0.182	0.96 (0.72–1.27)	0.780
	No		294 (26.7)		1 [Reference]		664 (60.3)		1 [Reference]		719 (65.2)		1 [Reference]	
**Being previously active (attending gym regularly)**	Yes		155 (50.5)	**<0.001**	3.60 (2.71–4.80)	<**0.001**	220 (71.7)	**<0.001**	1.58 (1.17–2.12)	**0.003**	239 (77.9)	**<0.001**	1.83 (1.33–2.52)	**<0.001**
	No		223 (20.3)		1 [Reference]		614 (55.8)		1 [Reference]		667 (60.6)		1 [Reference]	

Abbreviations: AOR adjusted odds ratio; CI, confidence interval.

### 3.4 Changes in weight

Almost half of the sample (695; 49.4%) reported weight gain with 52.2% reporting a 3–6 kg average increase in weight since the start of staying at home measures. We used an ordinal logistic regression analysis to determine the predictors of weight gain taking into consideration the reported weight gain (no change, <3 kg, 3–6 kg, 7–10 kg, >10 kg) as the dependent ordinal variable. Two models were carried out: one to analyze the impact of sociodemographic characteristics on weight gain, and the second to analyze the impact of the number of unhealthy dietary behaviors adopted during staying at home measures, and the changes in time spent in exercise, sitting/reclining, and screen time (all adjusted for sociodemographic characteristics) on weight gain. Both models were of good fit and statistically significant (*χ2*_(18)_ 112.124, *p* < 0.001) and (*χ2*_(26)_ 491.69, *p* < 0.001) for the first and second models respectively. Approximately 8% and 32% (Nagelkerke R^2^) of the variance in the weight gain can be explained by the first and the second models respectively. In the first model, participants with nationalities of Middle Eastern-North African and Sub-Saharan African origins were more likely to fall in higher weight gain categories than those from nationalities of Asia-Pacific origin with (OR 1.88, CI_95%_ 1.43–2.48*, p* < 0.001) and (OR 1.83, CI_95%_ 1.08–3.07, *p =* 0.023) respectively. Those who used to go to the gym regularly before the staying at home measures were more likely to report higher weight gain than those who did not (OR 1.78, CI_95%_ 1.39–2.28, *p* < 0.001). On the other hand, participants aged 55–64 years were more likely to fall in lower weight gain categories compared to those aged 18–24 years (OR 0.41, CI_95%_ 0.19–0.88, *p* = 0.023). No significant associations were found between weight change and gender, employment status (whether working from home or not), or history of chronic diseases (see Table 5).

**Table 5:** Sociodemographic predictors of increased body weight during COVID-19 related “staying at home” measures using ordinal logistic regression analysis (model 1).

Variable			No (%) of participants in each weight gain category					AOR (95%CI)	*p*-value
			No increase in weight	<3 kg	3–6 kg	7–10 kg	>10 kg		
Age	18–24 (n = 57)		21 (36.8)	12 (21.1)	16 (28.1)	4 (7.0)	4 (7.0)	1 [Reference]	
	25–34 (n = 540)		243 (45.0)	81 (15.0%)	163 (30.2)	36 (6.7)	17 (3.1)	0.88 (0.501–1.55)	0.658
	35–44 (n = 544)		291 (53.5)	73 (13.4)	133 (24.4)	36 (6.6)	11 (2.0)	0.65 (0.36–1.15)	0.135
	45–54 (n = 203)		117 (57.6)	27 (13.3)	40 (19.7)	16 (7.9)	3 (1.5)	0.55 (0.29–1.03)	0.061
	55–64 (n = 59)		37 (62.7)	8 (13.6)	11 (18.6)	1 (1.7)	2 (3.4)	0.41 (0.19–0.88)	**0.023**
	65+ (n = 5)		4 (80.0)	1 (20.0)	0 (0.0)	0 (0.0)	0 (0.0)	0.15 (0.01–1.07)	0.098
Gender	Male (n = 828)		463 (55.9)	100 (12.1)	193 (23.3)	50 (6.0)	22 (2.7)	0.82 (0.65–1.05)	0.119
	Female (n = 580)		250 (43.1)	102 (17.6)	170 (29.3)	43 (7.4)	15 (2.6)	1 [Reference]	
Nationality (classification by regions)	Americas (n = 45)		18 (40.0)	10 (22.2)	13 (28.9)	4 (8.9)	0 (0.0)	1.30 (0.74–2.29)	0.356
	Europe (n = 117)		46 (39.3)	27 (23.1)	27 (23.1)	13 (11.1)	4 (3.4)	1.40 (0.95–2.08)	0.092
	Sub-Saharan Africa (n = 54)		21 (7.0)	7 (13.0)	19 (35.2)	5 (9.3)	2 (3.7)	1.83 (1.08–3.07)	**0.023**
	Middle East – North Africa (n = 273)		116 (42.5)	20 (7.3)	86 (31.5)	35 (12.8)	16 (5.9)	1.88 (1.43–2.48)	**<0.001**
	Asia – Pacific (n = 919)		512 (55.7)	138 (15.0)	218 (218)	36 (3.9)	15 (1.6)	1 [Reference]	
Highest degree of education	No formal education (n = 18)		13(1.0)	1 (5.6)	2 (11.1)	1 (5.6)	1 (5.6)	0.35 (0.10–1.10)	0.083
	High school diploma (n = 228)		144 (63.2)	24 (10.5)	40 (17.5)	13 (5.7)	7 (3.1)	0.48 (0.27–0.86)	**0.012**
	College or Higher (n = 1107)		531 (48)	171 (15.4)	303 (27.4)	74 (6.7)	28 (2.5)	0.68 (0.41–1.15)	0.144
	Vocational training (n = 55)		25 (45.5)	6 (10.9)	18 (32.7)	5 (9.1)	1 (1.8)	1 [Reference]	
Employment status during “staying at home” measures	Employed n = 1070	Work from home (n = 568)	279 (49.1)	80 (14.1)	152 (26.8)	41 (7.2)	16 (2.8)	1.02 (0.76–1.37)	0.891
		Work regularly (n = 502)	284 (56.6)	62 (12.4)	125 (24.9)	25 (5.0)	6 (1.2)	0.78 (0.58–1.10)	0.163
		Not employed (n = 338)	150 (44.4)	60 (17.8)	86 (25.4)	27 (8.0)	15 (4.4)	1 [Reference]	
Marital status	Married (n = 1132)		592 (52.3)	159 (14.0)	281 (24.8)	77 (6.8)	23 (2.0)	1.05 (0.79–1.39)	0.745
	Not married (n = 276)		121 (43.8)	43 (15.6)	82 (29.7)	16 (5.8)	14 (5.1)	1 [Reference]	
Chronic disease	Yes (n = 306)		171 (55.9)	35 (11.4)	74 (24.2)	19 (6.2)	7 (2.3)	0.94 (0.72–1.22)	0.643
	No (n = 1102)		542 (49.2)	167 (15.2)	289 (26.2)	74 (6.7)	30 (2.7)	1 [Reference]	
Being previously active (attending gym regularly	Yes (n = 307)		109 (35.5)	53 (17.3)	103 (33.6)	30 (9.8)	12 (3.9)	1.78 (1.39–2.28)	**<0.001**
	No (n = 1107)		604 (54.9)	149 (13.5)	260 (23.6)	63 (5.7)	25 (2.3)	1 [Reference]	

Abbreviations: AOR adjusted odds ratio; CI, confidence interval.

In the second model, participants who reported a higher number of unhealthy dietary behavioral changes, were more likely to report greater weight gain (*p* < 0.001). For example, those who reported five unhealthy dietary changes were 13 times more likely to report higher weight gain categories compared to those who did not report any unhealthy changes (OR 13.41, CI_95%_ 8.56–21.02, *p* < 0.001). The mean reduction in exercise time, and the mean increases in sitting/reclining, and screen times were significantly associated with greater weight gain (see Table 6).

**Table 6:** Dietary behavioral and physical activity changes as predictors of weight gain during COVID-19 related “staying at home” measures using ordinal logistic regression analysis (Model 2).

Variable		No (%) of participants in each weight gain category					AOR* (95%CI)	*P*-value
		No increase in weight	<3 kg	3–6 kg	7–10 kg	>10 kg		
Number of unhealthy dietary behaviors	0 (n = 411)	332 (80.8)	39 (9.5)	33 (8.0)	5 (1.2)	2 (0.5)	1 [Reference]	
	1 (n = 271)	157 (57.9)	51 (18.8)	53 (19.6)	7 (2.6)	3 (1.1)	2.64 (1.87–3.72)	**<0.001**
	2 (n = 241)	107(44.4)	39 (16.2)	78 (32.4)	15 (6.2)	2(0.8)	4.71 (3.32–6.68)	**<0.001**
	3 (n = 197)	61(31.0)	31 (15.7)	75 (38.1)	23 (11.7)	7 (3.6)	7.78 (5.37–11.27)	**<0.001**
	4 (n = 180)	35 (19.4)	26 (14.4)	81 (45.0)	26 (14.4)	12 (6.7)	13.32 (9.04–19.63)	**<0.001**
	5 (n = 108)	21 (19.4)	16 (14.8)	43 (39.8)	17 (15.7)	11 (10.2)	13.41 (8.56–21.02)	**<0.001**
		Mean time difference for each weight gain category						
Exercise time difference^†^ (Hours/day)		0.12	0.00	0.43-	0.68-	0.66-	0.78 (0.70–0.86)	**<0.001**
Sitting/reclining time difference^†^ (Hours/day)		1.29	1.89	2.56	3.48	5.03	1.04 (1.01–1.08)	**0.026**
Screen time Difference^†^ (Hours/day)		1.46	1.70	2.74	3.67	4.35	1.06 (1.02–1.10)	**0.003**

Abbreviations: AOR, adjusted odds ratio; CI, confidence interval.

* Adjusted for variables in Table 5.

†Calculated as time during “staying at home” measures minus time before “staying at home” measures.

## 4 Discussion

Despite the benefits gained from the strict measures, including movement restrictions and staying at home orders in mitigating the spread of the COVID-19 infection, adverse health consequences have occurred. In this population-based study, we explored the impact of staying at home measures on dietary behaviors, physical activity, and body weight. The sample in this study is somehow representative of the wider population in Qatar in terms of the age distribution and the ethnic origin of participants because 93.7% of Qatar’s population above 24 years are between 25 and 54 years and 4.7% are between 55 and 64 years consistent with our study findings (Planning and Statistic Authority, 2021, Qatar – The World Factbook, 2021). Additionally, over 70% are non-Arabs similar to our study in which the non-Arabs comprised 80.6% of the total sample (Population of Qatar by nationality, 2019). On the other hand, the sex ratio in our sample (male: female ratio of 1.4:1) does not match the one in the true population (male: female ratio of 3.4:1) (Qatar – The World Factbook, 2021). Moreover, 78.6% of the study participants had a college degree or higher contrary to Qatar’s population where only 29% of those 15 years and above have a similar education level (Planning and Statistics Authority, 2019).

Regarding the overall perception of changes in dietary behaviors, nearly a third reported that their diet became less healthy since the start of the staying at home measures. This is consistent with a study in the United States where 31% of the participants reported worsened overall dietary behaviors (Khubchandani et al., 2020). Participants reported several unhealthy changes in their diet such as eating more fatty food and more sugar/sweets consistent with other studies conducted in neighboring Gulf countries such as Kuwait (Husain and Ashkanani, 2020), and the United Arab of Emirates (UAE) (Ismail et al., 2020). However, about one third of the participants in our study reported eating more fast-junk food unlike the studies in Kuwait and UAE where participants had reported drastic decreases in such food consumption. The switch to less healthier food choices, which are high in fat and sugar among the participants, may be attributed to emotional eating behaviors (eating due to stress or boredom). In fact, almost three quarters attributed such behaviors to increased feelings of boredom during home confinement and COVID-19 related stress. The evolving literature is currently shedding some light on the growing problem of emotional eating behaviors during the Covid-9 pandemic (Khubchandani et al., 2020, Rodgers et al., 2020, Al-Musharaf, 2020). On the other hand, one study that assessed food choice determinants during the COVID-19 pandemic had shown a decrease in perception of the importance of mood as a determinant which may promote the intake of a diet of higher nutritional value (Głąbska et al., 2020).

Although a high proportion of our participants reported healthy dietary behavioral changes such as consuming more fruits and vegetables (75.4%), and depending more on home cooking (63.1%), most of them reported at least one additional unhealthy dietary change. This is consistent with the findings of a recent scoping review that concluded that the lockdown due to Covid-19 results in both favorable and non-favorable dietary behaviors (Bennett et al., 2021). In our study, females, those of younger ages, and those unmarried reported higher numbers of unhealthy dietary changes similar to other studies (Allabadi et al., 2021, Deschasaux-Tanguy et al., 2019). We believe that unmarried individuals in Qatar are less likely to cook at home and depend on ordering food from restaurants that had continued providing services without interruption during the pandemic. Furthermore, lack of family support to these individuals during the pandemic might have been a source of stress resulting in adverse dietary behaviors, such as emotional eating.

This study, as well, showed a significant reduction in physical activity and an increase in sedentary behaviors expressed in terms of reduction in the time spent in exercise, and increase in sitting/reclining and screen times similar to other studies (Ammar et al., 2020, Robinson et al., 2021, Schnitzer et al., 2020 Aug, Brancaccio et al., 2021, Ismail et al., 2020, Husain and Ashkanani, 2020, Zachary et al., 2020, Górnicka et al., 2020). It is of paramount importance for people to maintain physical activity during staying at home measures. Evidence has shown that maintaining moderate intensity exercises is immunoenhancing rendering the human body more resistant to infections such as the COVID-19 (Scudiero et al., 2021). The reduction in exercise time in our study was minor, yet statistically significant for those who used to be active and were going to the gym regularly before the pandemic. Those who were not previously active showed a non-significant minor increase in their exercise time. On the other hand, studies in Austria (Schnitzer et al., 2020) and Belgium (Constandt et al., 2020) showed significantly higher exercise levels and more engagement in sport activities among previously less active people during the lockdowns. The reduction in exercise might be related to an inability to adapt to training at home after gym closures, lack of exercise equipment, or limited space at homes to allow performing different forms of exercise. Furthermore, the hot and humid weather in Qatar during summertime when restrictions were in place might have caused people reluctant to participate in outdoor exercises when permitted. Two thirds of our participants reported a significant increase in screen time. One possible explanation may be that distant operation of schools, offices and other institutions might have resulted in an increased use of digital devices as a way of interpersonal communication. Moreover, during staying at home measures, many health care institutions began providing virtual, instead of face-to-face, consultations with patients. As well, being forced to stay at home might have impelled people to spend more time in front of televisions or mobile devices for leisure purposes. Another reason for such increased time might have been a need to closely follow global events related to the pandemic. Lastly, with the closure of shopping malls and many retail stores, online shopping became the only means for consumers to satisfy their consumption needs.

Regarding the changes in body weight, a higher proportion of participants (49.4%) in our study perceived weight gain since the start of staying at home measures compared to other studies (Ismail et al., 2020, Zachary et al., 2020, Keel et al., 2020). This finding is alarming and could worsen existing obesity figures in Qatar and it signals a potential epidemic of lifestyle related non-communicable diseases in the post COVID-19 era. According to a report published by Qatar Biobank, the prevalence of obesity and overweight in Qatar is 43%, and 35%, respectively (Qatar Biobank Annual Report 2018/2019). The reduction in exercise time, the increase in sitting/reclining and screen times, and the number of unhealthy dietary changes were found to be significant predictors of weight gain in this study. These findings replicate the results of other studies that reported similar associations (Zachary et al., 2020, Reyes-Olavarría et al., 2020). However, the female gender was found to be a predictor of weight gain in other studies, but not in ours (Reyes-Olavarría et al., 2020, Đogaš et al., 2020, Di Renzo et al., 2020).

The indirect long term health consequences of COVID-19 might be more dangerous than its direct effects and constitute a potentially major public health concern. The emergence of different SARS-CoV-2 variants that appeared initially in the United Kingdome, South Africa, and the recent Delta variant in India after a period of relative viral genetic stability is concerning and can lead to a severe epidemic rebound (Fontanet et al., 2021). In fact, authorities in various countries are racing to contain the spread of the highly contagious Delta variant and are reimposing lockdown measures after periods of lifting restrictions (COVID, 2021). The ability of the SARS-CoV-2 to mutate in ways that accelerates virus transmission and reduces vaccine effectiveness, vaccine hesitancy, and the naïve assumptions about herd immunity, given the appearance of new and challenging SARS-CoV-2 variants, could seriously result in repeated outbreak recurrences (Skegg et al., 2021). History of previous pandemics and infectious outbreaks tells us that COVID-19 will not be the last pandemic, particularly with continued population growth, climate change, globalization and international travel that serve as triggers for emerging infections (Thoradeniya and Jayasinghe, 2021). As COVID-19 continues to spread and movement restriction measures are applied, the promotion of healthy lifestyles becomes increasingly more critical to prevent adverse health impacts. Additionally, researchers need to focus on addressing the persistence of such negative repercussions on health in the post pandemic era. It is worth noting that now it is the right time for countries to invest in introducing physical literacy among their populations which is a multidimensional concept that encompasses cognitive, emotional and social components that have a mutually beneficial relationship with physical activity. Physical literacy improves people’s confidence, knowledge and understanding of the importance of engaging in physical activity for life (Shahidi et al., 2020).

### 4.1 Strengths and limitations

This study had several strengths. As a population study, we were able to recruit an acceptable sample size of 1408 participants. The survey was distributed in four languages which may be considered a point of strength in a multilingual country like Qatar. In addition, this study was one of the few conducted in the Middle East to address this important area of research.

We also acknowledge that this study had several limitations. First, the sampling technique we used might have introduced selection bias affecting the representativeness of the sample. Second, the measurements obtained (such as exercise, screen and setting/reclining times, and weight gain) were self-reported and liable to information bias. Third, we did not use a validated questionnaire as we could not find a questionnaire that assesses all the aspects of lifestyle we needed, all together at the time of conducting this study. Fourth, while the questionnaire was developed based on a thorough literature review, its reliability was not assessed. Additionally, our conclusion about adverse changes in physical activity is mainly based on the reduction in exercise times and the increase in sedentary behaviors. However, other forms of physical activities that we did not assess such as housework might have increased. So physical activity related results should be interpreted with caution. Lastly, having to compare dietary behaviors and physical activity with those prior to staying at home measures might result in recall bias. However, at the time of conducting this study, using web-based self-reported survey was the only means of addressing this area of research in the light of the physical distancing measures recommended during this pandemic.

## 5 Conclusion

The results of this study indicate that Qatar’s population experienced adverse lifestyle changes with regard to their diet and physical activity. About 50% reported weight gain which is an alarming finding that can precipitate future epidemics of lifestyle related non-communicable diseases and worsen an already growing problem of obesity in Qatar. Although staying at home measures are critically important to mitigate the spread of COVID-19 infection, the findings of this study should be taken into consideration for future regulations in Qatar. It is of paramount importance to encourage people to maintain healthy lifestyle behaviors during staying at home measures that might be imposed during any public health crises or any potential future outbreaks. Evidence from this study should further stimulate efforts directed toward assessing and following up patients’ lifestyle related indices by healthcare workers and guide the implementation of effective lifestyle related interventions.

## CRediT authorship contribution statement

**Muna Abed Alah:** Conceptualization, Methodology, Project administration, Writing – original draft. **Sami Abdeen:** Methodology, Writing – original draft. **Vahe Kehyayan:** Supervision, Writing - review & editing. **Iheb Bougmiza:** Supervision, Writing - review & editing.

## Declaration of Competing Interest

The authors declare that they have no known competing financial interests or personal relationships that could have appeared to influence the work reported in this paper.
